# Supplementary material for: Genome-wide association studies reveal distinct genetic correlates and increased heritability of antimicrobial resistance in Vibrio cholerae under anaerobic conditions
Source: Microb Genom. 2022 Dec 5;8(12):mgen000905. doi: 10.1099/mgen.0.000905 (PMC9837564; doi:10.1099/mgen.0.000905)
Supplement: Supplementary material 1 [file mgen-8-905-s001.pdf]

SUPPLEMENTARY MATERIAL

Table of Contents

Supplementary Results. 2

Supplementary Table 1. *Reference strains and clinical isolates* 4

Supplementary Table 2. *Baseline growth parameters of V. cholerae clinical isolates under aerobic and anaerobic conditions* 6

Supplementary Table 3. *Growth and pH metrics with and without 20mM fumarate supplementation.* 7

Supplementary Table 4. *Minimal inhibitory concentrations (MICs) for ciprofloxacin, azithromycin, and doxycycline among V. cholerae clinical isolates* 8

Supplementary Table 5. *Comparison of rates of resistance detected under aerobic versus anaerobic conditions among V. cholerae clinical isolates in the primary collection* 9

Supplementary Table 6. *Comparison of rates of resistance detected under aerobic versus anaerobic conditions among V. cholerae clinical isolates in the secondary collection* 9

Supplementary Table 7. *Effect of catalase on growth parameters for V. cholerae E7946 and EN160 under aerobic conditions* 10

Supplementary Table 8. *Comparison of antibiotic resistance phenotypes and known resistance genotypes among V. cholerae clinical isolates in the primary collection* 11

Supplementary Table 9. *Test of association between antibiotic detection by mass spectrometry and AMR genotypes and phenotypes among V. cholerae clinical isolates* 12

Supplementary References 13

Supplementary Data Files (excel file with four datafile tabs)

Date File S1. GWAS Hits. Ciprofloxacin resistance phenotype. Aerobic.
Date File S2. GWAS Hits. Ciprofloxacin resistance phenotype. Anaerobic.
Data File S3. GWAS Hits. Azithromycin resistance phenotype. Anaerobic.
Data File S4. GWAS Hits. Doxycycline resistance phenotype. Anaerobic.

Supplementary Results.

Antibiotic resistance phenotypes of known AMR genetic elements under aerobic and anaerobic conditions. The distribution of known AMR genetic elements (Figure 2) was grouped by point mutations (likely transmitted vertically, not on an established mobilizable element) and horizontal transmission (on an established mobilizable element).

Point mutations in known AMR genes. For ciprofloxacin, the most identified known resistance mutations were those in the topoisomerase encoding genes *gyrA* (VC1258/RS06370) (Ser83Ile) and *parC* (VC2430/RS12340) (Ser85Leu). These were present in 60% (40/67) and 70% (47/67) of all isolates, while 54% (36/57) contained both. For azithromycin, we found no mutations in known resistance genes encoding ribosomal proteins L4 (*rplD;*VC2595/RS13175) and L22 (*rplV;*VC2591/RS13155) [1, 2]. For tetracycline (proxy for doxycycline), one of the 9 total 16S rRNA genes (VCr001) was found to have a single nucleotide insertion of a G nucleotide at position 327 within the sequences of 15% (10/67) of isolates; the significance is unknown. No mutations were detected in the 30S ribosomal protein encoded by genes *rpsJ* (VC2597/RS13185) or *rpsC* (VC2590/RS13150), whose mutations have been associated with tetracycline class resistance in other Gram negative organisms [3-5].

Known AMR genes on mobile genetic elements. The integrative conjugative element (ICE) SXT/R391 was found in 90% (60/67) of isolates. The ICE contained a pentapeptide repeat protein conferring fluoroquinolone resistance (qnr_{Vc}), a macrolide-inactivating phosphotransferase (*mphA*), and a major facilitator superfamily (MFS) efflux pump conferring tetracycline resistance (*tet*(59)) [6-9]. The genes qnr_{Vc}, *mphA*, and

tet(59) were also found in 78% (52/67), 33% (22/67), and 78% (52/67) of isolates, respectively.

Supplementary Table 1. Reference strains and clinical isolates

		Aerobic[b]			Anaerobic[b]			Source[c]
	Strain[a]	CIP^R	AZI^R	DOX^R	CIP^R	AZI^R	DOX^R	
Reference	E7946	-	-	-	-	+	-	(1)
Clinical Isolates								
EN018	L_EN1286	-	-	-	-	+	-	(2)
EN026	L_EN1291	-	-	-	-	+	-	(2)
EN027	L_EN1292	-	-	-	-	+	-	(2)
EN071	L_EN1300	+	-	-	+	+	-	(2)
EN072	L_EN1301	-	-	-	+	+	-	(2)
EN078	L_EN1303	+	-	-	+	+	-	(2)
EN079	L_EN1304	-	+	-	+	+	-	(2)
EN080	L_EN1305	+	-	-	+	+	-	(2)
EN086	L_EN1307	+	+	-	+	+	-	(2)
EN088	L_EN1308	+	-	-	+	+	-	(2)
EN092	L_EN1310	+	-	-	+	+	-	(2)
EN095	L_EN1312	+	+	-	+	+	-	(2)
EN096	L_EN1313	+	+	-	+	+	-	(2)
EN100	L_EN1314	+	+	-	+	+	-	(2)
EN103	L_En1315	-	-	-	+	+	-	(2)
EN109	L_EN1319	-	-	-	+	+	-	(2)
EN116	L_EN1325	-	-	-	+	+	-	(2)
EN117	L_EN1326	-	-	-	-	+	-	(2)
EN118	L_EN1327	-	+	-	+	+	-	(2)
EN119	L_EN1328	+	-	-	+	+	-	(2)
EN120	L_EN1329	-	-	-	+	+	-	(2)
EN123	L_EN1330	-	-	-	+	+	-	(2)
EN124	L_EN1331	+	-	-	+	+	-	(2)
EN125	L_EN1332	+	+	-	+	+	-	(2)
EN126	L_EN1333	+	-	-	+	+	-	(2)
EN127	L_EN1334	+	-	-	+	+	-	(2)
EN129	L_EN1335	-	-	-	+	+	-	(2)
EN130	L_EN1336	+	-	-	+	+	-	(2)
EN131	L_EN1337	+	-	-	+	+	-	(2)
EN132	L_EN1338	+	-	-	+	+	-	(2)
EN133	L_EN1339	+	-	-	+	+	-	(2)
EN134	L_EN1340	-	+	-	+	+	-	(2)

EN135	L_EN1341	-	-	-	+	+	-	(2)
EN137	L_EN1343	+	+	-	+	+	-	(2)
EN141	L_EN1344	+	-	-	+	+	-	(2)
EN143	L_EN1346	-	-	-	+	+	-	(2)
EN144	L_EN1347	+	-	-	+	+	-	(2)
EN145	L_EN1348	+	+	-	+	+	+	(2)
EN146	L_EN1349	+	-	-	+	+	-	(2)
EN147	L_EN1350	-	-	-	+	+	-	(2)
EN148	L_EN1351	-	-	-	+	+	-	(2)
EN149	L_EN1352	+	-	-	+	+	-	(2)
EN150	L_EN1353	-	-	-	+	+	-	(2)
EN153	L_EN1355	-	-	-	+	+	-	(2)
EN155	L_EN1357	+	-	-	+	+	-	(2)
EN156	L_EN1358	-	-	-	+	+	-	(2)
EN159	L_EN1360	+	+	-	+	+	-	(2)
EN160	L_EN1361	+	-	-	+	+	-	(2)
EN162	L_EN1363	+	+	-	+	+	-	(2)
EN164	L_EN1365	-	-	-	+	+	-	(2)
EN165	L_EN1366	+	-	-	+	+	-	(2)
EN166	L_EN1367	+	-	-	+	+	-	(2)
EN167	L_EN1368	+	-	-	+	+	-	(2)
EN168	L_EN1369	+	+	-	+	+	-	(2)
EN169	L_EN1370	-	-	-	+	+	-	(2)
EN171	L_EN1371	+	+	+	+	+	+	(2)
EN173	L_EN1372	-	-	-	+	+	-	(2)
EN174	L_EN1374	-	-	-	+	+	-	(2)
EN178	L_EN1377	+	-	-	+	+	-	(2)
EN181	L_EN1379	-	-	-	-	+	-	(2)
EN182	L_En1380	-	-	-	+	+	-	(2)
EN183	L_EN1381	-	-	-	+	+	-	(2)
EN184	L_EN1382	+	-	-	+	+	-	(2)
EN185	L_EN1383	-	-	-	+	+	-	(2)
EN188	L_EN1385	-	-	-	+	+	-	(2)
EN189	L_EN1386	+	+	-	+	+	-	(2)
EN191	L_EN1388	-	-	-	+	+	-	(2)

[a] Prefix of “L_” was used to distinguish the strain number in the library from the clinical isolate number, which is maintained to be consistent with prior publications.

[b] CIP^R = ciprofloxacin resistance. AZI^R = azithromycin resistance. DOX^R = doxycycline resistance.

[c] Sources of strains: (1) Mekalanos, J. J. Duplication and amplification of toxin genes in Vibrio cholerae. Cell 35, 253-263, (1983) ; (2) Nelson, E. J. et al. Complexity of rice-water stool from patients with Vibrio cholerae plays a role in the transmission of infectious diarrhea. Proc Natl Acad Sci U S A 104, 19091-19096, (2007).

Supplementary Table 2. Baseline growth parameters of *V. cholerae* clinical isolates under aerobic and anaerobic conditions

Growth parameter [a]	Aerobic median	Anaerobic median	P [b]
K	1.08	0.261	**< 0.001**
AUC	5.15	1.58	**< 0.001**
Velocity	0.011	0.006	**< 0.001**

[a] AUC = area under the curve (optical density x time in minutes). K = carrying capacity. Media was LB alone without antibiotics. Velocity = growth rate in percent increase per minute at half the carrying capacity.
[b] Wilcoxon signed-rank test for growth. Bold = statistically significant (P<0.05).

Supplementary Table 3. Growth and pH metrics with and without 20mM fumarate supplementation.

		Anaerobic				Aerobic			
		Replicate 1		Replicate 2		Replicate 1		Replicate 2	
Strain	Fumarate	AUC[a]	pH[b]	AUC[a]	pH[b]	AUC[a]	pH[c]	AUC[a]	pH[c]
**E7946**	**-**	1.63	7.2	1.53	7.2	5.11	7.2	5.42	7.2
	**+**	1.93	7.2	1.85	7.2	5.33	7.2	5.52	7.2
**EN145**	**-**	1.28	6.4	1.22	6.4	4.96	7.2	5.10	7.2
	**+**	1.65	7.2	1.55	7.2	4.98	7.2	5.33	7.2
**EN160**	**-**	1.55	6.8	1.33	6.8	4.63	7.2	5.03	7.2
	**+**	1.92	7.2	1.66	7.2	5.03	7.2	5.23	7.2
**EN181**	**-**	1.25	**6.0**	1.20	**6.0**	5.02	7.2	5.10	7.2
	**+**	1.65	6.8	1.63	7.2	5.38	7.2	5.30	7.2
Average (st.dev)	**-**	1.43 (0.19)	…	1.32 (0.15)	…	4.93 (0.21)	…	5.16 (0.17)	…
	**+**	1.79 (0.16)	…	1.67 (0.12)	…	5.18 (0.21)	…	5.35 (0.12)	…
Percent difference with fumarate		+25%	…	+27%	…	+5.1%	…	+3.7%	…

[a] 'AUC' = area under the curve (optical density x time in minutes).
[b] Bold numbers represents pH of test wells below the pH of the LB blank control well at the end of the assay (pH 6.4); the pH of the LB blank control well at the start of the assay was 6.8.
[c] The starting and ending pH of the LB control blank wells were 6.8 and 7.2 for both replicates, respectively.

Supplementary Table 4. Minimal inhibitory concentrations (MICs) for ciprofloxacin, azithromycin, and doxycycline among *V. cholerae* clinical isolates

	Ciprofloxacin[b]		Azithromycin[b]		Doxycycline[b]	
μg/ml[a]	Aerobic	Anaerobic	Aerobic	Anaerobic	Aerobic	Anaerobic
0.002	0	---	---	---	---	---
0.004	1	---	---	---	---	---
0.008	0	---	---	---	---	---
0.016	2	1	0	---	0	0
0.032	0	0	0	0	0	0
0.063	0	1	0	0	0	0
0.13	0	1	0	0	2	1
0.25	0	0	0	0	7	4
0.5	5	0	0	0	14	9
1	23	2	5	0	**38**	**26**
2	**35**	4	22	0	2	21
4	0	1	**23**	0	3	4
8	1	**56**	15	3	1	1
16	---	0	0	4	0	1
32	---	1	2	**44**	0	0
64	---	---	---	14	---	---
124	---	---	---	2	---	---

[a] Concentration of antibiotic.
[b] Distribution of MICs for clinical isolates grown under aerobic and anaerobic conditions. Bold text signifies the concentration at which the MIC mode was determined among the clinical isolates.

Supplementary Table 5. Comparison of rates of resistance detected under aerobic versus anaerobic conditions among *V. cholerae* clinical isolates in the primary collection

	N	R^{Ae}/R^{An}	R^{Ae}/S^{An}	S^{Ae}/R^{An}	S^{Ae}/S^{An}	p[b]
Ciprofloxacin[a]	67	36	0	26	5	**<0.001**
Azithromycin[a]	67	15	0	52	0	**<0.001**
Doxycycline[a]	67	1	0	1	65	1

[a] Distribution of paired resistant ('R') and sensitive ('S') phenotypes for isolates under aerobic ('Ae') and anaerobic ('An') conditions. R is defined as an MIC at or above the CLSI breakpoints (see methods).
[b] McNemar's Exact Test. Bold = statistically significant (P<0.05)

Supplementary Table 6. Comparison of rates of resistance detected under aerobic versus anaerobic conditions among *V. cholerae* clinical isolates in the secondary collection

	N	R^{Ae}/R^{An}	R^{Ae}/S^{An}	S^{Ae}/R^{An}	S^{Ae}/S^{An}	p[b]
Ciprofloxacin[a]	277	2	1	56	218	**<0.001**
Azithromycin[a]	277	159	0	118	0	**<0.001**
Doxycycline[a]	277	1	0	3	273	0.371

[a] Distribution of paired resistant ('R') and sensitive ('S') phenotypes for isolates under aerobic ('Ae') and anaerobic ('An') conditions. R is defined as an MIC at or above the CLSI breakpoints (see methods).
[b] McNemar's Exact Test. Bold = statistically significant (P<0.05)

Supplementary Table 7. Effect of catalase on growth parameters for *V. cholerae* E7946 and EN160 under aerobic conditions

Experiment	Strain[a]	Antibiotic[b]	Catalase[c]	AUC mean[d]	AUC IQR[d]	P[e]
1.	E7946	CIP	YES	0.697	0.029	0.136
		CIP	NO	0.736	0.014	
2.	E7946	AZI	YES	3.26	0.056	0.533
		AZI	NO	3.29	0.005	
3.	E7946	DOX	YES	5.14	0.105	0.959
		DOX	NO	5.14	0.070	
4.	E7946	NO	YES	5.29	0.078	0.818
		NO	NO	5.30	0.090	
5.	EN160	CIP	YES	2.31	0.368	0.551
		CIP	NO	2.10	0.420	
6.	EN160	AZI	YES	4.43	0.119	0.571
		AZI	NO	4.50	0.113	
7.	EN160	DOX	YES	4.67	0.322	0.895
		DOX	NO	4.60	0.360	
8	EN160	NO	YES	5.33	0.188	0.092
		NO	NO	5.44	0.146	

[a] E7946 (Cip^{S}, Azi^{S}, Dox^{S}) is the reference strain and EN160 (Cip^{R}, Azi^{R}, Dox^{S}) is a clinical isolate. Biological replicates in experiments 1-3 and 5-7 were 3, each with 4 technical replicates. Biological replicates for experiments 4 and 8 were 9, each with 4 technical replicates.

[b] CIP = ciprofloxacin. AZI = azithromycin. DOX = doxycycline. Assays were run at CIP = 0.5, AZI = 2, and DOX = 0.25 µg/ml for EN160; E7946 was run at CIP = 0.002, AZI = 1, and DOX = 0.013 µg/ml.

[c] CIP and AZI were tested with 3 biological replicates; DOX with 2 biological replications; LB controls with 9 biological replicates.

[d] AUC = area under the curve. IQR = interquartile range.

[e] Student’s t-test. Bold = statistically significant ($P<0.05$)

Supplementary Table 8. Comparison of antibiotic resistance phenotypes and known resistance genotypes among *V. cholerae* clinical isolates in the primary collection

Aerobic conditions [a]

Antibiotic tested	Gene	R/P	R/NP	S/P	S/NP	OR[b]	95% CI[b]	P[b]
Ciprofloxacin	*qnr*$_{Vc}$	36	0	16	15	31	(4.22 - 695)	**<0.001**
	gyrA	28	8	12	19	5.4	(1.86 - 17.9)	**0.002**
	parC	31	5	16	15	5.6	(1.65 - 18.9)	**0.003**
Azithromycin	*mphA*	15	0	7	45	83	(11.2 - 1938)	**<0.001**
Doxycycline	*tet*(59)	1	0	51	15	0.62	(0.046 - 18.9)	0.566

Anaerobic conditions [a]

Antibiotic tested	Gene	R/P	R/NP	S/P	S/NP	OR[b]	95% Cb[c]	P[b]
Ciprofloxacin	*qnr*$_{Vc}$	52	10	0	5	27	(3.53 - 666)	**<0.001**
	gyrA	40	22	0	5	10	(1.39 - 248)	**0.016**
	parC	47	15	0	5	17	(2.28 - 416)	**0.003**
Azithromycin	*mphA*	22	45	0	0	0.51	(0.0127 - 20.1)	1
Doxycycline	*tet*(59)	1	0	51	15	0.62	(0.046 - 18.9)	0.566

[a] Whole genome sequencing data from the primary collection were analyzed for known AMR genes. R = resistant phenotype, S = sensitive phenotype, P = gene present, NP = gene not present. Enumerations include isolates with at least the specific gene named; isolates may have more than one resistance gene (e.g. *qnr*$_{Vc}$, *gyrA* and *parC*).

[b] Fisher's Exact Test. Bold = statistically significant ($P<0.05$)

Supplementary Table 9. Test of association between antibiotic detection by mass spectrometry and AMR genotypes and phenotypes among *V. cholerae* clinical isolates

Antibiotic detection (D) and AMR genotype present (P) [a]					
	D/P	D/NP	ND/P	ND/NP	*P*[b]
CIP	41	7	3	0	1
CIP + NAL	35	4	9	3	0.334
DOX	16	8	20	7	0.759
DOX + TET	2	0	34	15	1
**Antibiotic detection (D) and resistance phenotype (R) under aerobic conditions [a]**					
	D/R	D/S	ND/R	ND/S	*P*[b]
CIP	24	24	3	0	0.238
CIP + NAL	22	17	5	7	0.511
DOX	1	23	0	27	0.471
DOX + TET	0	2	1	48	1
**Antibiotic detection (D) and resistance phenotype (R) under anaerobic conditions [a]**					
	D/R	D/S	ND/R	ND/S	*P*[b]
CIP	44	4	3	0	1
CIP + NAL	38	1	9	3	**0.036**
DOX	1	23	0	27	0.217
DOX + TET	0	2	1	48	1

[a] D=detected, ND=not detected, R=resistant by MIC, S=sensitive by MIC, P = Present, NP = Not present.
[b] Fisher's Exact Test. Bold = statistically significant ($P<0.05$)

Supplementary References

1. **Davydova N, Streltsov V, Wilce M, Liljas A, Garber M**. L22 ribosomal protein and effect of its mutation on ribosome resistance to erythromycin. *J Mol Biol* 2002;322(3):635-644.
2. **Hansen LH, Mauvais P, Douthwaite S**. The macrolide-ketolide antibiotic binding site is formed by structures in domains II and V of 23S ribosomal RNA. *Mol Microbiol* 1999;31(2):623-631.
3. **Williams G, Smith I**. Chromosomal mutations causing resistance to tetracycline in *Bacillus subtilis*. *Mol Gen Genet* 1979;177(1):23-29.
4. **Wei Y, Bechhofer DH**. Tetracycline induces stabilization of mRNA in *Bacillus subtilis. J Bacteriol* 2002;184(4):889-894.
5. **Beabout K, Hammerstrom TG, Perez AM, Magalhaes BF, Prater AG et al.** The ribosomal S10 protein is a general target for decreased tigecycline susceptibility. *Antimicrob Agents Chemother* 2015;59(9):5561-5566.
6. **Leclercq SO, Wang C, Zhu Y, Wu H, Du X et al.** Diversity of the Tetracycline Mobilome within a Chinese Pig Manure Sample. *Appl Environ Microbiol* 2016;82(21):6454-6462.
7. **Grossman TH, Starosta AL, Fyfe C, O'Brien W, Rothstein DM et al.** Target- and resistance-based mechanistic studies with TP-434, a novel fluorocycline antibiotic. *Antimicrob Agents Chemother* 2012;56(5):2559-2564.
8. **Noguchi N, Emura A, Matsuyama H, O'Hara K, Sasatsu M et al.** Nucleotide sequence and characterization of erythromycin resistance determinant that encodes macrolide 2'-phosphotransferase I in *Escherichia coli*. *Antimicrob Agents Chemother* 1995;39(10):2359-2363.
9. **Chesneau O, Tsvetkova K, Courvalin P**. Resistance phenotypes conferred by macrolide phosphotransferases. *FEMS Microbiol Lett* 2007;269(2):317-322.
